# Utilizing Haddon matrix to assess nonfatal commercial fishing injury factors in Oregon and Washington

**DOI:** 10.1186/s40621-023-00428-7

**Published:** 2023-03-24

**Authors:** Solaiman Doza, Viktor Bovbjerg, Samantha Case, Amelia Vaughan, Laurel Kincl

**Affiliations:** 1 College of Public Health and Human Sciences, 14A Milam Hall, Oregon State University, Corvallis, OR 97330 USA; 2 National Institute for Occupational Safety and Health, Western States Division, Anchorage, AK USA

**Keywords:** Fishermen, Risk factors, Injury events, Work processes, Injury prevention

## Abstract

**Background:**

Commercial fishing is a precarious industry with high fatal and nonfatal injury rates. The Risk Information System of Commercial [RISC] Fishing project at Oregon State University has been tracking both fatal and nonfatal injuries among Oregon and Washington commercial fishermen. We examined the utility of the RISC dataset variables in highlighting injury factors and prevention opportunities.

**Method:**

We identified 245 nonfatal commercial fishing injuries in Oregon and Washington (2000–2018) and assessed the top three injury events (contact with objects or equipment, transportation incidents, and slips/trips/falls) using a cross-sectional design. We generated a Haddon matrix for each event type and populated the matrices with injury-associated factors following our a-priori matrix.

**Results:**

We observed 108 nonfatal injuries due to contact with objects. Contact injuries occurred during fishing (40%) with fishing gears (40%), often while hauling the fishing gear (22%). Common injury mechanisms included getting caught in running equipment or machinery (19%) or compressed by shifting objects or equipment (18%). Of the 58 transportation injuries most occurred in catchers (93%) and smaller vessels (1 to 3 crew) (55%). Vessel casualties were common as several vessels struck rocks/bottom (29%) or experienced fire and explosion (19%). The crew was abandoned to water (38%), often due to no raft or raft malfunctions (19%). Slip/trip/fall injuries (*n* = 43) typically happened during onboard traffic (49%). Such events were largely experienced by the catcher-processors (44%) including large vessels with > 100 crew (28%).

**Conclusion:**

The Haddon matrix demonstrated the injury-event timeline and helped to identify potential injury-associated factors. Our injury-specific risk matrices will let commercial fishing stakeholders determine priorities and work with the experts on prevention efforts.

**Supplementary Information:**

The online version contains supplementary material available at 10.1186/s40621-023-00428-7.

## Background

Commercial fishing has one of the highest fatal and nonfatal injury burdens in the US industries and comprises unique work-specific risk factors (i.e. drowning) (Bovbjerg et al. 2019; Case et al. 2018; Lincoln and Lucas 2010a; Lucas and Lincoln 2007). Injury prevention efforts primarily focus on events leading to the death of commercial fishermen but in recent years, further emphasis has also been given to nonfatal injury prevention (CDC - Commercial Fishing Safety: Fishing Safety Research Projects - NIOSH Workplace Safety and Health Topic 2020; Commercial Fishing Safety 2015). Common nonfatal fishing injuries include sprains/strains, surface wounds, cuts, punctures, and fractures (Bovbjerg et al. 2019; Case et al. 2015). Several previous studies showed that primary prevention strategies following a public health approach were effective in both fatal and nonfatal injury reduction (Issa et al. 2019; Lehtola et al. 2008; Lincoln et al. 2017; Lincoln and Conway 1999; Lucas et al. 2014; Stout and Linn 2002). A study by Lincoln et al. (2017) successfully designed standardized passive guards to prevent winch entanglements and recommended utilizing injury epidemiologic methods and industry input to design effective safety interventions (Lincoln et al. 2017). Lucas et al. (2014) evaluated a novel safety policy intervention by the United States Coast Guard (USCG) and found a successful reduction of vessel disaster incidents and improved worker safety (Lucas et al. 2014). To promote injury prevention, Dr. William Haddon Jr established a logical framework (known as the Haddon matrix) to systematically identify key injury factors (Haddon et al. 1964; Haddon 1968, 1972). The matrix is based on the epidemiologic triangle (Host, agent, and environment) and is widely utilized to evaluate injury events and identify preventive measures (Susser 1973). Previously, National Institute for Occupational Safety and Health (NIOSH) utilized the Haddon matrix to identify risk factors and countermeasures for commercial fishing fatalities in Alaska (Lincoln and Conway 1997). Commercial fishing events for fatal injuries are qualitatively different compared to nonfatal injury cases. Moreover, injury risk factors can vary depending on fishery type, vessel type, vessel activities, crew activities, and injury location (Bovbjerg et al. 2019; Lincoln and Lucas 2010a, b; Lincoln et al. 2021; Jin and Thunberg 2005; Thomas et al. 2001; Jin et al. 2001; Jensen 2000). Hence, nonfatal injury factors should be explored for an improved understanding of overall commercial fishing hazards. NIOSH tracks commercial fishing incidents and fatalities in a nationwide database—Commercial Fishing Incident Database (CFID) (Case et al. 2018; Lucas and Case 2018; Commercial Fishing Safety: Research Projects | NIOSH | CDC 2021). Yet, nonfatal fishing injury events can be missed due to passive data collection methods (Bovbjerg et al. 2019). We collaborated with NIOSH to improve the CFID database and include the nonfatal commercial fishing injuries through an ongoing data project (Risk Information System of Commercial [RISC] Fishing) at Oregon State University (RISC 2019). Currently, the RISC database comprises commercial fishing fatalities, vessel casualties, and nonfatal injury cases. We evaluated the nonfatal injury cases in Oregon and Washington from the RISC database to identify potential injury factors at the host, agent, physical, and social environmental cells of the Haddon matrix. Oregon and Washington have a rich and diverse fishing industry with a variety of vessels that range from small catcher vessels with generally 1–6 crew onboard to large processing vessels (i.e., catcher-processor or processor) with dozens of crewmembers used for industrial fishing operations (Oregon’s Commercial Fishing in 2021; Commercial fishing|Washington Department of Fish Wildlife 2023). Catcher vessels typically utilize fishing techniques including trawling, longlining, and gillnetting to catch fish whereas processing vessels, which may or may not harvest seafood, are larger vessels that process fish at sea in their onboard factories. The fishing fleets in Oregon and Washington play a crucial role in the economy and culture of the Pacific Northwest and ongoing monitoring and research are necessary to ensure their sustainability and productivity (Observed Fishing Effort and in the U.S. Pacific Coast Groundfish Fisheries: At-Sea Midwater Trawl Catcher-Processor 2023). Our analysis explored the utility of the RISC dataset variables in highlighting injury factors and prevention opportunities to minimize nonfatal fishing injuries in the region. The proposed work generated individual Haddon matrices to describe each of the top three presenting injury events populated with nonfatal injury data from Oregon and Washington from the RISC Fishing database.

## Materials and methods

### Case definition, data source, and study design

Our study focused on nonfatal injuries sustained by commercial fishermen in Oregon and Washington between 2000 and 2018, as reported to the 13th District of the United States Coast Guard (USCG) (the region bounded by Canada to the north and California to the south) (U.S. Coast Guard 2022a). We utilized the nonfatal injury dataset and the vessel casualty dataset from the RISC database and identified 245 nonfatal injury cases (with or without vessel disasters) which were originally recorded in the CFID database by NIOSH. Through an interagency Memorandum of Agreement, NIOSH routinely has access to the USCG form CG-2692 to identify commercial fishing incident cases. The CG-2692 incident reports include the following: (a) intentional or unintentional grounding; (b) loss of propulsion or primary steering; (c) loss of seaworthiness or fitness for service; (d) loss of life; (e) injury which requires treatment beyond first aid and renders the individual unfit to perform routine duties; and (f) damage exceeding $25,000 (U.S. Coast Guard 2022b). We worked with NIOSH to manually abstract the nonfatal incident data, from the electronically stored scanned copies of the CG-2692 forms, which were then entered into both the CFID and RISC databases (Nahorniak et al. 2021).

We used a cross-sectional study design and combined multi-year nonfatal injury data to generate overall percentages of injury-associated factors, as there were insufficient cases to produce annual injury proportions.

### Injury-event-specific Haddon matrices

We created individual Haddon matrices for each of the three event types using an a-priori conceptual Haddon matrix. The a-priori matrix consisted of all possible injury-associated factors recorded in the RISC database. These factors were tabulated according to the injury event type to determine the utility of RISC dataset variables in informing the Haddon matrix. (Table 1). The RISC Fishing project has organized an advisory board composed of experts in occupational injury and health safety experts, public health researchers, a USCG vessel safety coordinator, representatives of fishing industry groups, marine fishery experts of the National Oceanic and Atmospheric Administration (NOAA), members of the Commercial Fishing Safety Advisory Committee, and other field experts with relevant knowledge and experience (Vaughan et al. 2022). During the August 2021 meeting, we presented our a-priori matrix to the advisory board members and requested their feedback on potential key contributing factors and related safety priorities. This collaborative effort enabled us to develop a robust and comprehensive a-priori matrix that addresses stakeholder needs.

**Table 1:** A-priori Haddon matrix of nonfatal injury associated factors in commercial fishing (RISC-data variables are in parentheses)

Phase	Host	Agent/vehicle	Physical environment	Social environment
Pre-event	Hazardous work task (Position, work process). Lack of safety knowledge (Safety training, train year) Fishing/working under the influence (Drugs, alcohol) Medical conditions (Preexisting condition) Lack of PFD use (PFD worn) Fatigue (Incident notes)	Potential injury vehicles (Incident type, injury source, gear type, injury event, cause of instability, type of flooding, cause of flooding, final vessel event, initial injury event, incident notes)	Vessel activity during the incident (Vessel activity, vessel type, Fishery type, species) Vessel capacity (People onboard) Housekeeping status (Incident notes) Adverse weather conditions (Weather-related, sky condition, wind speed, air temp, and water temp) Light conditions (Incident time, incident notes) Physical infrastructure (Vessel length/size, hull material, year built) Safety equipment status (PFD location donned, incident notes) Vessel/equipment maintenance (Incident notes)	Federal/state/regional fishing safety compliance (USCG Decal, EPIRB present, PFD policy, fishery jurisdiction) Language barriers (English fluency, primary language) Safety training status (Safety training, train year, incident notes, PFD policy)
Event	The task associated with injury (Position, work process) Human error (Incident notes) Workload status (Incident notes) Crew experience and expertise (Age, year’s fishing) Safety gear use (PFD worn, abandon to, why water, incident notes) Lack of situational awareness (Incident notes) Panic response (Incident notes)	Injury vehicle (Injury source, injury event, incident notes) Injury severity (Nature of injury, body part injured, injury severity, incident notes)	Injury location (Location onboard, abandon to, incident notes) Vessel location (Latitude, longitude, miles from shore, vessel activity) Safety equipment status (PFD type, why water, incident notes)	Language barriers (English fluency, primary language) Safety training status (Safety training, train year, incident notes, PFD policy)
Post-event	Injury response activity (Injury response, incident notes) Injury treatment description (Injury treatment, incident notes)	Improved vessel maintenance (Final vessel event, cause of instability, type of flooding, cause of flooding, fire cause, fire location, incident notes)	Delayed injury response (Mayday method, miles from shore, incident notes) Adverse weather conditions (Weather-related, sky condition, wind speed, air temp, and water temp) Light conditions (Incident time, incident notes)	Revised regulations and safety measures (USCG Decal, EPIRB present, PFD policy, safety training, train year, incident notes)

The analysis using the Haddon matrix yielded potential risk factors at different stages/time points (pre-event, during-event, and post-event). The top three injury events (contact with objects or equipment, transportation incidents, and slips/trips/falls) were identified by tabulating the frequencies of the Occupational Injury and Illness Classification System (OIICS) event category variable. NIOSH utilized the standard OIICS classification in their CFID database to group the commercial fishing injury events, but on occasion modified the OIICS rules to assign codes that better reflect the onboard injury event. For example, injury events due to contact with objects or equipment were coded in the relevant subcategory instead of coding as “water vehicle incident” as suggested in the OIICS manual (oiics_manual_2010 2021). The OIICS data dictionary defined transportation injury events as injuries that occurred due to vessel casualty or disaster, fall or jump overboard, machinery or equipment failure, and explosion or fire (OIICS Code Trees 2023). NIOSH also used a modified version of the OIICS classification to accurately represent the injury sources (Syron et al. 2016). The injury severity was recorded in both CFID and RISC databases based on the Maximum Abbreviated Injury Scale (MAIS) by the Association for the Advancement of Automotive Medicine (AAAM) (Abbreviated Injury Scale (AIS) 2023).

In the result matrices, we presented the top two percentage categories (the tables are included as Additional file 1: tables) for each contributing factor with a few exceptions. We included the third and fourth categories if they were equal or similar in proportion to the second group (≤ 2% difference). Continuous variables were recoded into categories. The number of crew members was categorized as 1–3, 4–9, 10–99, and ≥ 100. The number of work years of the injured crewmember was grouped as < 1, 1–3, 4–10, and > 10. Fishermen's age was grouped as 18–29, 30–39, 40–49, and ≥ 50. We presented all categories for these demographic variables to illustrate the injured worker distribution. We recorded it as unknown if the variable had any of the following categories—null, unknown, unknown in source, unclassifiable, and missing. Variables with large proportions of missing or unknown values (≥ 90%) were excluded. For instance, variables such as sky condition, wind speed, air temperature, and water temperature were excluded from the result matrices. Primary language and English fluency both had high missing values and were excluded.

## Theory

**Fig. 1:**
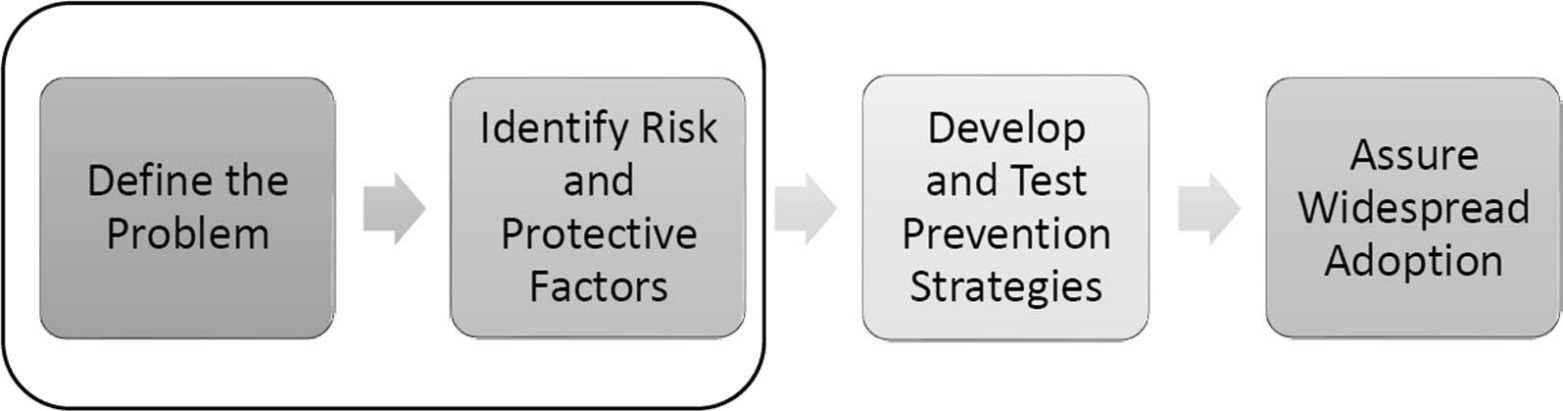
Public health model

The Centers for Disease Control and Prevention (CDC) recommends utilizing a public health model for injury prevention efforts. This is a systematic process that has been widely used for disease prevention through the steps shown in Fig. 1 (Our Approach|Injury Center|CDC 2020).

Our current work addresses the first and second steps of the public health model. First, we utilized existing surveillance data to define the work-associated injuries among Oregon and Washington commercial fishermen. Second, we evaluated the usefulness of the nonfatal injury records using the Haddon matrix (a framework based on injury event timeline and the epidemiologic triangle host, agent, and environment) (Haddon 1972). This is a solution-oriented model which has been vastly applied for injury prevention to reduce morbidity and mortality from a variety of injury types. The framework allows for the development of strategies to implement primary, secondary, and tertiary preventive measures at different phases of the injury event (Lucas et al. 2014; Christoffel and Gallagher 2006). Human factors are grouped under the host column. The injury agents such as any thermal, electrical, or mechanical energy that can affect the host via an injury vehicle are categorized as agent factors under the agent column. The physical environment column includes the physical features of the location where the injury event takes place (i.e., fishing vessel, agriculture farm, forest ground). The social environment column consists of the social and legal norms and practices such as fishing practices in a particular fishing community.

## Results

Of the 245 nonfatal commercial fishing injury cases, 44% (*n* = 108) were due to contact with objects or equipment, 24% (*n* = 58) were transportation incidents, and 18% (*n* = 43) occurred through a slip/trip/fall. Overexertion (5%; *n* = 11) and harmful substance exposure (4%) were the other two leading event types. The most relevant factors, determined by frequency, for each injury event type (contact with objects or equipment, transportation incidents, and slips/trips/falls) are shown in the corresponding Haddon matrices (Tables 2, 3, 4).

**Table 2:** Haddon matrix of factors related to contact with objects/equipment injuries (*n* = 108) in Oregon and Washington fisheries

Phase	Host	Agent/vehicle	Physical environment	Social environment
Pre-event	Position: *Deckhand & Processor* Work process: *Hauling gear, Handling gear on deck, processing catches,* &* handling frozen fish*	Injury source: *Fishing gear* & *processing equipment* Gear type: *Trawl* & *Pot/trap* Injury event: *Caught in running equipment or machinery during regular operations* &* compressed or pinched by shifting objects or equipment*	Vessel activity: *Fishing & transit* Vessel type: *Catcher* & *catcher and processor* Fishery type: *Groundfish & Shellfish* Species: *Dungeness Crab & Hake* Crew size: *1 to 3 crew, 4 to 9 crew,* & ≥ *100 crew*	USCG Decal: *Current*
Event	Injured worker positions: *Deckhand & processor* Work process: *Hauling gear, Handling gear on deck, Processing catches,* &* Handling frozen fish* Age group:*18 to 29 years & 40 to 49 years* Years fishing: *4 to 10 years &* > *10 years*	Injury source: *Fishing gear* & *processing equipment* Injury event: *Caught in running equipment or machinery during regular operation* &* compressed or pinched by shifting objects or equipment* Injury nature: *Fractures & other open wounds* Injury body part: *Upper extremity & lower extremity* MAIS Injury Severity: *Minor & moderate*	Location onboard: *Deck/stern deck/bow deck & outrigger* Vessel activity: *Fishing & transit*	
Post-event	Injury response: *USCG evacuation & vessel returned* Injury treatment: *Hospital treatment & compression/pressure*			

The most common factors are provided for each variable applicable to each cell. See full descriptive statistics for each variable in Additional file 1: Tables

**Table 3:** Haddon matrix of factors related to transportation injuries (*n* = 58) in Oregon and Washington fisheries

Phase	Host	Agent/vehicle	Physical environment	Social environment
Pre-event	Position: *Owner/operator/skipper & Deckhand* Work process: *Watch, working in engine room, off-duty* Drug: *Not suspected & Marijuana* Alcohol: *Tested & suspected* Human factors: *None & alcohol consumption*	Incident type: *Vessel casualty* Injury source: *Substance and environment* Initial vessel disaster event: *Struck Rocks/Bottom & Smoke/Fire/Explosion* Injury event: *Capsized or sinking vessel & Explosion or fire on vessel*	Vessel activity: *Transit & fishing* Vessel type: *Catcher* Fishery type: *Shellfish & Pelagic fish* Species: *Dungeness Crab & Salmon* Gear type*: Pot/trap & Troll* Crew size: *1 to 3 crew & 4 to 9 crew* Weather-related: *Yes*	Fishery Jurisdiction: *Tribal & STATE* USCG Decal: *Expired & none* Safety training: *Training received (i.e., NPFVOA, AMSEA)*
Event	Position: *Owner/operator/skipper & Deckhand* Work process: *Watch, Working in engine room, Off-duty* Age group: *40 to 49 years* Year’s fishing: > *10 years* PFD worn: No	Injury source: *Substance and environment* Injury nature: *Effects of environmental conditions & burns and corrosions* Injured body part: *Body systems* MAIS Injury Severity: *Minor & moderate*	Location onboard: *Wheelhouse & Deck* Abandon to: *Water & other vessels* Why in water: *Swam to shore & no raft present* PFD type: *Immersion suit*	Safety training: *Training received (i.e., NPFVOA, AMSEA)*
Post-event	Injury response: *Recovered by other vessel &USCG evacuation* Injury treatment: *No treatment*	Final vessel event: *Grounded & sinking* Flooding type: *Below water line*	Mayday method: *Radio & cell phone* EPIRB present: *Yes* Abandon to: *Water & other vessels*	USCG Decal: *Expired & none* Safety training: *Training received (i.e., NPFVOA, AMSEA)*

The most common factors are provided for each variable applicable to each cell. See full descriptive statistics for each variable in Additional file 1: Tables

**Table 4:** Haddon matrix of factors related to slip/trip/fall injuries (*n* = 43) in Oregon and Washington fisheries

Phase	Host	Agent/vehicle	Physical environment	Social environment
Pre-event	Position: *Processor & Deckhand* Work process: *Traffic on board* Alcohol: *Tested* PFD worn: *No*	Injury-source: *Fishing vessel* Gear type: *Trawl*	Vessel activity: *Moored & transit* Fishery type: *Groundfish* Species: *Pollock & Pacific Whiting/Hake* Vessel type: *Catcher and processor & Catcher* Crew size: *4 to 9 crew &* ≥ *100 crew*	USCG Decal: *Current*
Event	Position: *Processor & Deckhand* Work process: *Traffic on board* Age group: *18 to 29 years & 30 to 39 years* Year’s fishing: *1 to 3 years* PFD worn: *No*	Injury-source: *Fishing vessel* Gear type: *Trawl * Injury nature: *Fractures & surface wounds and bruises* Injury body part: *Lower extremity & multiple body parts* MAIS Injury Severity: *Minor & serious*	Location onboard: *Deck* Vessel activity: *Moored & Transit* Vessel crew size: *4 to 9 crew &* *≥* *100 crew*	
Post-event	Injury response: *Moored—treated in a clinic* *USCG evacuation, &treated on vessel* Injury treatment: *Advanced clinic/hospital treatment*			USCG Decal: *Current*

The most common factors are provided for each variable applicable to each cell. See full descriptive statistics for each variable in Additional file 1: Tables

We tabulated 54 factors (RISC Fishing data variables) for our result matrices, of which 15 were host-related, 14 were agents, 19 were physical environments, and 6 were considered in the social column of the a-priori matrix (Table 1). Of these factors 19 were excluded from the result matrices as they had a large proportion (≥ 90%) of missing or unknown responses; 35 factors were used to generate the three result matrices for the top three injury events (contact with objects or equipment, transportation incidents, and slips/trips/falls). Ten factors were included in the host column, nine were in the agent column, thirteen were in the physical environment column, and three were in the social environment column (Tables 2, 3, 4).

Among the 108 fishermen injured due to contact with objects or equipment about half (49%) were working as deckhands. Most vessels were catcher (51%), or catcher-processor (38%), and were harvesting groundfish (53%), or shellfish (29%). Fishing gear (40%) was the dominant injury source followed by processing equipment (22%). The fishermen typically suffered contact injuries while they were actively fishing (40%), often due to hauling the fishing gear (22%). Upper extremities (62%) were the dominant body part injured and many of these injuries occurred due to getting caught in running equipment or machinery (19%) or compressed by shifting objects or equipment (18%). The injury severity was mostly moderate (46%) but 19% were serious in nature (MAIS severity categories) (Data not presented in the Haddon matrices; please see Additional file 1: Tables S1, S2).

Transportation injuries (*n* = 58) equally affected all crew positions and of the injured nearly half (45%) were vessel owners/operators/skippers and another 43% were deckhands (Table 3). The majority of transportation injury events (93%) happened on catcher vessels, and the prevalence was higher on smaller vessels carrying 1–3 crew members (55%). The dominant fishery type was shellfish (64%), and the majority species was the Dungeness crab (52%). Vessel casualties or disasters often occurred as the vessel struck rocks/bottom (29%) or suffered fire or explosion (19%) and about one-third (33%) were capsized. Several vessels had crews working under the influence as 14% tested positive for marijuana and 12% were suspected of alcohol use. Adverse weather conditions contributed to 14% of the transportation injury events. Nearly one-third (29%) of the injury events occurred under tribal jurisdiction. The crew often abandoned to water (38%) due to no raft or raft malfunctions (19%). About one-third (31%) of the injured wore personal flotation devices (PFDs). Burns and corrosions (26%) and injuries resulting from adverse environmental conditions (35%) were the two most common types of injuries, with more than 40% of injury severity falling between moderate and severe. Crews were generally recovered by other vessels (29%) and USCG (28%) (See Additional file 1: Tables S1, S2).

Slip/trip/fall injuries (*n* = 43) commonly occurred during onboard traffic (49%) and processing the catch (16%). Injured workers largely comprised processors (30%) and deckhands (28%) (Table 4). The dominant vessel type that suffered a slip/trip/fall injury was catcher-processors (44%) including large vessels with ≥ 100 crew (28%). Most vessels (79%) were harvesting groundfish which included species like pollock (30%), and pacific whiting/hake (26%). The top nature of injury were fractures (23%), and lower extremities (33%) were the dominant injured body part. Nearly one-third (28%) suffered serious injuries (See Additional file 1: Tables S1, S2).

## Discussion

### Haddon matrix application

A Haddon matrix helps to systematically identify priorities for prevention and control at different time points of the injury event (pre-event, event, and post-event). Our findings highlighted that the top injury events among the Oregon and Washington commercial fishermen had varied contributing factors that may require distinct prevention efforts. The contact with objects injury events commonly occurred during operating or handling fishing gear, or processing and handling the catch. In contrast, transportation injuries typically resulted from vessel casualties, and the crew often suffered body system injuries from exposure to cold seawater, fire, or smoke. Slip/trip/fall injuries commonly occurred while walking or moving on the vessel. Fractures were common in both contact injuries and slip/trip/falls injuries, yet the upper extremity was largely affected by contact with objects whereas the lower extremity was mostly affected by falling onboard the vessel. An earlier report by NIOSH analyzed the fatal commercial fishing injury events with the Haddon matrix and found that fatality rates varied depending on the types of the fishery, harvesting equipment and techniques, time of year, and length of the fishing season (Lincoln and Conway 1997; Conway et al. 1999). A study in Canada also utilized the Haddon matrix to conceptualize the mechanism of fall overboard fishing fatalities and identified example countermeasures for prevention (Tremblay et al. 2021). Our results demonstrated that the RISC Fishing database yielded adequate data points to populate the Haddon matrix but there are other potential factors (e.g., Vessel maintenance, fishing equipment maintenance, sea conditions) that are missing and need to be included to illustrate the injury pathway. The injury event characteristics can guide the prevention efforts and here we discuss a few potential interventions at different time points (pre-event, event, and post-event) of the injury event.

### Contact with objects or equipment injury events

During pre-event, contact with object injuries can be reduced through engineering controls such as installing enclosures over running machines, and safety guards on sharp processing equipment to modify the injury agents. Specialized safety training may focus on the catcher vessel crew regarding work tasks, such as hauling gear, handling gear on deck, handling heavy loads, using sharp equipment, and securing loose equipment or objects. Successful utilization of safety controls on the fishing gear and processing equipment can prevent severe injuries, especially during entanglement with gear or running machinery. The physical work environment can be improved through good housekeeping (e.g., securing stacked boxes and heavy equipment). Many of the contact injuries occurred due to a flying or falling object, which could be prevented by conducting routine checkups of potential unsecured objects or equipment as well as pre-sailing checks for frayed or worn lines. Effective workplace communication can help to improve the social environment and avert injuries due to coworker actions.

During contact injury events, social and host factors like teamwork, effective communication, and active assistance will help to minimize injury severity. Personal protective equipment (PPE) availability and use can greatly reduce injury severity during the event. For example, wearing safety gloves can reduce injury severity and help to free the hand when caught in running equipment or machinery. During the post-event, the duration between the injury and receiving treatment largely influences the recovery. A trained crew on first aid and preservation techniques of an injured body part can help to deter lifelong disability. During post-event, the availability and utilization of emergency contact equipment and routine safety drills can help in the prompt evacuation of the injured crew member.

### Transportation injury events

Transportation injuries largely occurred due to vessel casualties and hence the pre-event focus can be on improved navigation through updated equipment, operational skills, appropriate decision-making, and vessel maintenance. An earlier study explored human and organizational factors in maritime accidents and suggested decision errors as the key contributing factor resulting from poor visibility and misuse of instruments (environmental factors) as well as loss of situational awareness (human factor) (Chauvin et al. 2013). Our study could not explore such factors as the RISC dataset currently does not contain the supporting data. Small catcher vessels and vessels operating in the tribal jurisdiction disproportionately suffered transportation injury events. Hence, future prevention efforts need to target this high-risk group for prevention. Fire and explosion on the vessels might be due to poor vessel maintenance resulting in electrical short-circuit, improper or faulty repairing of electrical wiring, gas buildup in confined poorly ventilated spaces, fuel line leaks, and improper storage of extra fuel barrels (Jin et al. 2001; National Research Council (U.S.) 1991; ). Pre-event countermeasures may include routine pre-sailing checkups of the fuel lines and ventilation systems, and identifying leaks in hoses and piping systems to initiate prompt repairing and maintenance to reduce the risk of offshore vessel fires (USCG 2006).

Injury prevention during a transportation incident will require easy access to, and use of safety gear like rafts, PFDs, or fire protective coveralls. Active fire detection systems and portable fire-fighting equipment would also help to prevent transportation injury severity during the event (USCG 2006; Perez-Labajos 2008). The post-event intervention will include access to emergency contact equipment (e.g., very high frequency [VHF] marine radio, emergency position indicating radio beacon [EPIRB]) and necessary training for successful use during emergencies. Safety gear needs to be checked before leaving the port to ensure functionality. Availability and use of EPIRB, and personal locator beacons (PLBs) with the survival gear will ensure a rapid and effective response from USCG and other nearby vessels, particularly in events where early communication of distress is not possible.

### Slip/trip/fall injury events

Slip/trip/fall injuries were common during onboard traffic and often on large vessels. This could be because vessels with large crews may comprise busy traffic areas with high work demand. Spills or other hazards on the walkways can also result in a slip/trip/fall. Ladders and open holds or hatches could be another potential source of onboard injuries. For pre-event prevention, busy traffic areas can be marked and regulated by safety lines to control traffic flows. Ladders and other high places may include fitted handrails and traction tapes can be applied on both ladder steps and high-traffic areas to prevent slips/trips/falls. Pre-event use of slip-resistant shoes and routine cleaning of the spills can also prevent slip/trip/fall injury events.

During the event, the use of PPE like knee pads, safety goggles, and hard hats can reduce injury severity. Post-event prevention for slip/trip/fall injuries can be similar to other injury events as it will comprise prompt emergency communication, application of first aid, and swift evacuation efforts to ensure a full recovery.

### Limitations

This study had a few limitations, particularly those associated with the use of surveillance data, which may restrict our ability to identify certain patterns that could elucidate differences in the fishery, harvesting equipment, and techniques as previously studied concerning commercial fishing fatalities (Lincoln and Conway 1997, 1999; Conway et al. 1999). There are missed injury events as both CFID and RISC datasets only record injuries that require reporting to the USCG (Nahorniak et al. 2021). Bovbjerg et al. (2019), conducted an injury survey among the West coast Dungeness crab fishermen and found that most injuries did not require clinical care yet about half of the injuries reported limited their ability to work (Bovbjerg et al. 2019). Several key factors were either not recorded in the RISC database or recorded as missing/unknown. Some variables were only recently added to the database and thus not collected for older cases. For some factors, the desired information was not available from the USCG reports. Host factors like fatigue or excess workload were not possible to evaluate since it was not recorded. Physical environmental factors like sky condition, light condition, wind speed, air temperature, water temperature, and whether the condition of weather contributed to the injury event were present in the database, but the responses were largely missing or unknown. It may be possible, however, to merge existing databases such as ours with databases that document sea and weather conditions, to better understand their role in commercial fishing injuries. Social environmental factors such as primary language, and English fluency were also not documented, which could have shed light on language barriers and the scope of miscommunication between the fishing vessel workers. Inadequate language proficiency can also lead to a poor understanding of federal or state fishing regulations and weather advisories (Evangelos 2002; Levin et al. 2010, 2019). To identify additional factors related to contact with object injuries, incident narratives were examined by three authors (SD, VB, LK) using the a-priori matrix (Table 1). The lead author initiated the review and marked cases requiring consensus. The goal was to identify absent key factors among RISC dataset variables (e.g., Fatigue), but after review, no new factors were found. The narratives from the other two injury events were not reviewed due to their lower number of cases.

## Conclusions

Despite these drawbacks, the Haddon matrix largely helped organize the injury-event timeline and the observed factors associated with the nonfatal injury events in Oregon and Washington fisheries. Sharing these findings with workers and other experts (e.g., marine safety engineers, and commercial fishing safety regulators) may help identify a range of practical and effective injury prevention measures, designed to both prevent and ameliorate injuries. The injury-specific risk matrices will allow the commercial fishing stakeholders to determine priorities and work with the experts on prevention efforts. Future work can take these findings to further explore these frequent issues to determine targeted interventions.

## Practical applications

We will be sharing the result matrices on our web portal and promoting them through social media platforms to reach out and inform the commercial fishing stakeholders in Oregon and Washington. Based on these injury-associated factors we will work toward an evidence-based preventive strategy to minimize the injury burden, through our collaborations with our regional center, the Pacific Northwest Agricultural Safety, and Health Center.

## Supplementary Information

**Additional file 1. **Descriptive tables of the non-fatal commercial fishing injury factors for Oregon and Washington.

## Data Availability

The datasets generated and/or analyzed during the current study are not publicly available to assure privacy and confidentiality. We are unable to publish or share the dataset as the ethical approval of the study requires presenting the results in aggregate form to maintain participant anonymity. The study dataset was obtained from the Commercial Fishing Incident Database by NIOSH, and the Marine Information for Safety and Law Enforcement by the US Coast Guard which can be accessed through the agency's agreement process.

## Abbreviations

AAAM: Association for the Advancement of Automotive Medicine
CFID: Commercial Fishing Incident Database
EPIRB: Emergency position indicating radio beacon
MAIS: Maximum Abbreviated Injury Scale
MEDEVAC: Medical evacuation
NIOSH: National Institute for Occupational Safety and Health
NOAA: National Oceanic and Atmospheric Administration
OIICS: Occupational Injury and Illness Classification System
PFD: Personal flotation device
PLB: Personal locator beacon
PPE: Personal protective equipment
RISC Fishing: Risk Information System of Commercial Fishing
USCG: United States Coast Guard
VHF: Very high frequency
