# Supplementary material for: Utilizing Haddon matrix to assess nonfatal commercial fishing injury factors in Oregon and Washington
Source: Inj Epidemiol. 2023 Mar 24;10:18. doi: 10.1186/s40621-023-00428-7 (PMC10037792; doi:10.1186/s40621-023-00428-7)
Supplement: Supplementary file 1 — Additional file 1. Descriptive tables of the non-fatal commercial fishing injury factors for Oregon and Washington. [file 40621_2023_428_MOESM1_ESM.docx]

**Manuscript ID: INEP-D-23-00003**

**Title:** Utilizing Haddon matrix to assess nonfatal commercial fishing injury factors in Oregon and Washington

**Supplementary tables**

Supplemental Table 1. Event categories by the most common injury events among Oregon and Washington commercial fishing workers.*

| Contact with objects/equipment injury event (n = 108) | n (%) |
| --- | --- |
| *Caught in running equipment or machinery during regular operation* | 20 (18.5) |
| *Compressed or pinched by shifting objects or equipment* | 19 (17.6) |
| *Struck by falling object or equipment* | 18 (16.7) |
| *Contact with objects and equipment* | 10 (9.2) |
| *Struck by object or equipment* | 7 (6.5) |
| *Caught in or compressed by equipment or objects* | 5 (4.6) |
| *Struck by swinging or slipping object, other than handheld* | 5 (4.6) |
| Transportation injury event (n = 58) |  |
| *Capsized or sinking water vehicle* | 19 (32.8) |
| *Explosion or fire on water vehicle* | 17 (29.3) |
| *Collision between water vehicle and object* | 13 (22.4) |
| Slip/trip/fall injury event (n = 43) |  |
| *Fall to lower level* | 7 (16.3) |
| *Other fall to lower level* | 7 (16.3) |
| *Fall on same level due to tripping* | 7 (16.3) |
| *Slip without fall* | 6 (14.0) |

* variables with less than 5 cases are not shown to maintain anonymity.

Supplemental Table 2. Fishermen (host), agent and environment factors related to the top injury events for Oregon and Washington commercial fishing workers.**

|  | Contact with objects/equipment  n = 108 | Transportation injuries  n = 58 | Slip/trip/fall injuries  n = 43 |
| --- | --- | --- | --- |
| Age groups | n (%) | n (%) | n (%) |
| *18-29 years* | 31 (28.7) | <5 | 11 (25.6) |
| *30-39 years* | 15 (13.9) | <5 | 9 (20.9) |
| *40-49 years* | 23 (21.3) | <5 | 8 (18.6) |
| *>50 years* | 8 (7.4) | <5 | <5 |
| *Unknown* | 31 (28.7) | 46 (79.3) | 11 (25.6) |
| Position | | | |
| *Deckhand* | 53 (49.1) | 25 (43.1) | 12 (27.9) |
| *Processor* | 33 (30.6) | - | 13 (30.2) |
| *Owner-operator/skipper* | <5 | 26 (44.8) | <5 |
| *Unknown* | 12 (11.1) | 6 (10.3) | 10 (23.3) |
| Work process |  |  |  |
| *Hauling gear* | 24 (22.2) | - | <5 |
| *Handling gear on deck* | 12 (11.1) | <5 | - |
| *Processing the catch* | 12 (11.1) | - | 7 (16.3) |
| *Handling frozen fish* | 12 (11.1) | - | - |
| *Other work with the catch* | 7 (6.5) | - | - |
| *Traffic on board* | 6 (5.6) | - | 21 (48.8) |
| *Watch* | <5 | 23 (39.7) | - |
| *Working in engine room* | <5 | 7 (12.1) | <5 |
| *Off duty* | - | 7 (12.1) | <5 |
| *Unclassifiable* | - | 11 (19.0) | - |
| *Other* | 10 (9.3) | 6 (10.3) | 5 (11.6) |
| *Unknown* | 11 (10.2) | - | - |
| Years of fishing |  |  |  |
| *<1 year* | 10 (9.3) | <5 | <5 |
| *1 to 3 years* | 8 (7.4) | <5 | 8 (18.6) |
| *4 to 10 years* | 24 (22.2) | <5 | 7 (16.3) |
| *>10 years* | 22 (20.4) | 21 (36.2) | 7 (16.3) |
| *Unknown* | 44 (40.7) | 29 (50.0) | 17 (39.5) |
| Drug use |  |  |  |
| *Marijuana* | <5 | 8 (13.8) | <5 |
| *Not suspected* | 6 (8.3) | 12 (20.7) | 5 (11.6) |
| *Unknown* | 97 (89.8) | 37 (63.8) | 36 (83.7) |
| Alcohol use |  |  |  |
| *Tested* | 8 (7.4) | 17 (29.3) | 9 (20.9) |
| *Suspected* | - | 7 (12.1) | - |
| *Not suspected* | 5 (4.6) | 5 (8.6) | <5 |
| *Unknown* | 95 (88.0) | 29 (50.0) | 33 (76.7) |
| Human factors |  |  |  |
| *Alcohol Consumption* | - | 7 (12.1) | - |
| *Navigational Error* | - | 6 (10.3) | - |
| *None* | - | 16 (27.6) | - |
| *Unknown* | - | 23 (39.7) | - |
| Safety training |  |  |  |
| *Yes* | - | 12 (20.7) | - |
| *Unknown* | - | 42 (72.4) | - |
| PFD worn |  |  |  |
| *Yes* | - | 18 (31.0) | <5 |
| *No* | - | 18 (31.0) | 5 (11.6) |
| *Unknown* | - | 22 (37.9) | 37 (86.0) |
| Injury agent/source |  |  |  |
| *General tools and equipment* | 10 (9.3) | - | <5 |
| *Fishing gear* | 43 (39.8) | <5 | <5 |
| *Processing equipment* | 24 (22.2) | - | <5 |
| *Fishing vessel* | 18 (16.7) | 11 (19.0) | 30 (69.8) |
| *Fish and fish products* | 7 (6.5) | - | <5 |
| *Substances and environment* | - | 33 (57.9) | <5 |
| *Unknown* | <5 | 7 (12.1) | <5 |
| Injury location |  |  |  |
| *Deck/stern deck/bow deck* | 54 (50.0) | 6 (10.3) | 24 (55.8) |
| *Outrigger* | 15 (14.0) | - | 6 (14.0) |
| *In skiff* | 7 (7.0) | - | - |
| *Engine room* | <5 | 5 (8.6) | <5 |
| *Wheelhouse* | <5 | 7 (12.1) | - |
| *Unknown* | 21 (19.5) | 35 (60.3) | 6 (14.0) |
| Vessel Type |  |  |  |
| *Catcher* | 55 (50.9) | 54 (93.1) | 15 (34.9) |
| *Catcher/Processor* | 41 (38.0) | - | 19 (44.2) |
| *Processor* | 9 (8.3) | - | 8 (18.6) |
| *Unknown* | <5 | - | <5 |
| Fishery type |  |  |  |
| *Groundfish* | 57 (52.8) | <5 | 34 (79.1) |
| *Shellfish* | 31 (28.7) | 37 (63.8) | <5 |
| *Pelagic fish* | 13 (12.0) | 16 (27.6) | <5 |
| *Unknown* | 7 (6.5) | <5 | <5 |
| Species |  |  |  |
| *Dungeness Crab* | 24 (22.2) | 30 (51.7) | <5 |
| *Hake* | 20 (18.5) | - | <5 |
| *Pacific Whiting* | 18 (16.7) | <5 | 7 (16.3) |
| *Pollock* | 8 (7.4) | - | 13 (30.2) |
| *Shrimp* | 7 (6.5) | <5 | <5 |
| *Salmon* | 6 (5.6) | 11 (19.0) | - |
| *Sardine* | 5 (4.6) | - | - |
| *Cod/Black Cod (Sablefish)* | 5 (4.6) | - | <5 |
| *Tuna* | <5 | 5 (8.6) | <5 |
| *Unknown Groundfish* | <5 | - | 5 (11.6) |
| *Unknown* | 6 (5.6) | - | <5 |
| Fishing gear type |  |  |  |
| *Trawl* | 51 (47.2) | <5 | 25 (58.1) |
| *Pot/trap* | 25 (23.2) | 31 (53.5) | <5 |
| *No fishing gear* | 11 (10.2) | - | 8 (18.6) |
| *Seine* | 9 (8.3) | <5 | <5 |
| *Troll* | <5 | 9 (15.5) | <5 |
| *Drift/Set Gillnet* | - | 5 (8.6) | - |
| *Unknown* | <5 | <5 | <5 |
| Vessel activity |  |  |  |
| *Fishing* | 43 (39.8) | 20 (34.5) | 6 (14.0) |
| *Transit* | 31 (28.7) | 25 (43.1) | 14 (32.6) |
| *Moored* | 21 (19.4) | 7 (12.1) | 16 (37.2) |
| *Unknown* | 11 (10.2) | 5 (8.6) | 6 (14.0) |
| Vessel crew size |  |  |  |
| *1 to 3 crew* | 17 (15.7) | 32 (55.2) | <5 |
| *4 to 9 crew* | 19 (17.6) | 22 (37.9) | 8 (18.6) |
| *10 to 99 crew* | 11 (10.2) | - | <5 |
| *>100 crew* | 23 (21.3) | - | 12 (27.9) |
| *Unknown* | 38 (35.2) | <5 | 16 (37.2) |
| Injury nature |  |  |  |
| *Fractures* | 28 (25.9) | <5 | 10 (23.3) |
| *Amputations/avulsions/enucleations* | 26 (24.1) | - | - |
| *Other open wounds* | 28 (25.9) | <5 | <5 |
| *Surface wounds and bruises* | 10 (9.3) | 5 (8.6) | 9 (20.9) |
| *Sprains/strains/tears or other muscle/tendon/ligament/joint* | 5 (4.6) | <5 | 10 (23.3) |
| *Multiple traumatic injuries* | 5 (4.6) | <5 | <5 |
| *Effects of environmental conditions* | - | 20 (34.5) | - |
| *Burns and corrosions* | - | 15 (25.9) | - |
| *Unknown* | <5 | <5 | 5 (11.6) |
| Body parts injured |  |  |  |
| *Upper extremity* | 67 (62.0) | 6 (10.3) | 5 (11.6) |
| *Lower extremity* | 11 (10.2) | 5 (8.6) | 14 (32.6) |
| *Face and forehead* | 9 (8.3) | 7 (12.1) | <5 |
| *Multiple or unspecified head location* | 6 (5.6) | <5 | <5 |
| *Multiple body parts* | <5 | 13 (22.4) | 7 (16.3) |
| *Other body parts* | <5 | 5 (8.6) | <5 |
| *Body systems* | - | 16 (27.6) | - |
| MAIS Injury Severity |  |  |  |
| *Minor* | 33 (30.56) | 22 (37.9) | 15 (34.9) |
| *Moderate* | 50 (46.3) | 14 (24.1) | 10 (23.3) |
| *Serious* | 20 (18.5) | 10 (17) | 12 (27.9) |
| *Unknown* | 5 (4.6) | 9 (15.5) | 5 (11.6) |
| Initial vessel event |  |  |  |
| *Struck rocks/bottom* | - | 17 (29.3) | - |
| *Smoke/Fire/Explosion* | - | 11 (19.0) | - |
| *Flooding* | - | 5 (8.6) | - |
| *Unknown* | - | 12 (20.7) | - |
| Final vessel event |  |  |  |
| *Capsized or sinking water vehicle* | - | 19 (32.8) | - |
| *Explosion or fire on water vehicle* | - | 17 (29.3) | - |
| *Collision between water vehicle and object* | - | 13 (22.4) | - |
| Crew abandoned vessel to |  |  |  |
| *Water* | - | 22 (37.9) | - |
| *Other vessel* | - | 9 (15.5) | - |
| *Land* | - | 7 (12.1) | - |
| *Life raft* | - | 7 (12.1) | - |
| *Unknown* | - | 9 (15.5) | - |
| Abandoned to water |  |  |  |
| *No Raft Present/Raft malfunctioned/unreachable* | - | 11 (19.0) | - |
| *Swam to Shore* | - | 9 (15.5) | - |
| *Unknown* | - | 38 (65.5) | - |
| PFD type |  |  |  |
| *Immersion Suit* | - | 13 (22.4) | - |
| *Unknown* | - | 44 (75.9) | - |
| Weather-related |  |  |  |
| *Yes* | <5 | 8 (13.8) | <5 |
| *No* | 8 (7.4) | - | 5 (11.6) |
| *Unknown* | 98 (90.7) | 50 (86.2) | 37 (86.1) |
| Light condition |  |  |  |
| *Daylight* | 5 (4.6) | - | <5 |
| *Unknown* | 100 (92.6) | - | 38 (88.4) |
| Fishery jurisdiction |  |  |  |
| *Federal* | 5 (4.6) | <5 | <5 |
| *State* | - | 9 (15.5) | <5 |
| *Tribal* | <5 | 17 (29.3) | - |
| *Unknown* | 101 (93.5) | 31 (53.4) | 38 (88.4) |
| Decal status |  |  |  |
| *Current* | 23 (21.3) | 12 (20.7) | 15 (34.9) |
| *Expired* | 19 (17.6) | 15 (25.9) | <5 |
| *None* | <5 | 14 (24.1) | - |
| *Unknown* | 63 (58.4) | 17 (29.3) | 24 (55.8) |
| Injury response |  |  |  |
| *Evacuated by USCG helicopter/vessel* | 20 (18.5) | 16 (27.6) | 7 (16.3) |
| *Vessel immediately returned to shore* | 15 (13.9) | <5 | <5 |
| *Vessel moored and treated in clinic* | 10 (9.3) | 6 (10.3) | 10 (23.3) |
| *Received treatment on vessel* | 23 (21.3) | <5 | 10 (23.3) |
| *Recovered on shore by EMS* | <5 | 8 (13.8) | - |
| *Received by other vessel* | <5 | 17 (29.3) | - |
| *Unknown* | 37 (34.3) | 7 (12.1) | 11 (25.6) |
| Mayday method |  |  |  |
| *Emergency Position Indicating Radio Beacon (EPIRB)* | - | 5 (8.6) | - |
| *Cell phone* | <5 | 12 (20.7) | <5 |
| *Radio* | <5 | 21 (36.2) | <5 |
| *None* | 5 (4.6) | - | <5 |
| *Unknown* | 100 (92.6) | 16 (27.6) | 36 (83.7) |
| Emergency Position Indicating Radio Beacon (EPIRB) |  |  |  |
| *Yes* | - | 20 (34.5) | <5 |
| Injury treatment |  |  |  |
| *Advanced Clinic/Hospital Treatment* | 13 (12.0) | <5 | 5 (11.6) |
| *Compression/Pressure* | 7 (6.5) | <5 | - |
| *No treatment* | <5 | 6 (10.3) | <5 |
| *Unknown* | 77 (71.3) | 39 (67.2) | 33 (76.7) |

** To maintain anonymity, variables with less than 5 cases are indicated. In cases where all three events had less than 5 cases, variables are not shown.
